# Detection of volatile sulfur compounds (VSCs) in exhaled breath as a potential diagnostic method for oral squamous cell carcinoma

**DOI:** 10.1186/s12903-022-02301-3

**Published:** 2022-07-01

**Authors:** Ik-Jae Kwon, Tae-Young Jung, Youjeong Son, Bongju Kim, Soung-Min Kim, Jong-Ho Lee

**Affiliations:** 1 Department of Oral and Maxillofacial Surgery, Dental Research Institute, School of Dentistry, Seoul National University, 101 Daehakro, Jongnogu, Seoul, 03080 Korea; 2 Department of Oral and Maxillofacial Surgery, Busan Paik Hospital, Inje University, Bokji-ro 75, Busanjin-gu, Busan, 47392 Korea; 3 Dental Life Science Research Institute/Innovation Research & Support Center for Dental Science, Seoul National University Dental Hospital, Seoul, 03080 Korea

**Keywords:** Volatile sulfur compounds, Exhaled breath, Oral squamous cell carcinoma, Halitosis, Gas chromatography

## Abstract

**Background:**

Oral squamous cell carcinoma causes a significant proportion of global cancer morbidity and mortality. The aim of this study is to investigate whether the exhaled breath test can be a new, non-invasive, and effective method for diagnosing oral squamous cell carcinoma.

**Methods:**

A comparative analysis of exhaled breath between patients with oral squamous cell carcinoma (OSCC) and healthy controls (HC) was performed with the Twin Breasor II™, a simple gas chromatography system.

**Results:**

Both hydrogen sulfide (H_2_S) and methyl mercaptan (Ch_3_SH) were significantly higher in the OSCC group than in the HC group. The total sulfur concentration was also higher in the OSCC group, but there was no significant difference in the ratio of Ch_3_SH to H_2_S between the two groups. Using logistic regression, we constructed a new variable with an area under the curve (AUC) of 0.740, 68.0% sensitivity, and 72.0% specificity.

**Conclusions:**

Exhaled gas analysis via simple gas chromatography can potentially serve as an accessory non-invasive method for OSCC diagnosis.

**Supplementary Information:**

The online version contains supplementary material available at 10.1186/s12903-022-02301-3.

## Background

Oral cancer (OC) causes a significant proportion of global cancer morbidity and mortality. Worldwide, OCs account for 300,000 cases (2.1% of the world total) and 145,000 deaths per year [1]. Among these, oral squamous cell carcinoma (OSCC) represents 90% of all OCs and it commonly occurs in the oral cavity [2].

Despite great medical advances in recent decades to improve the prognosis of many cancers, the prognosis of OSCC remains poor [2, 3]. Clinical and histopathologic examination, in addition to imaging, are used to diagnose OSCC. Currently, invasive tissue biopsy followed by histopathologic examination is the gold standard for OSCC diagnosis [4]. However, tissue biopsy is an invasive procedure, and it takes 1 ~ 2 weeks to receive results. An early and simple test for diagnosing OSCC that can be performed non-invasively and yields immediate results could be an essential supplemental diagnostic tool. Diagnosis using exhaled gas is a promising non-invasive method. Changes in the composition of exhaled gas can cause oral malodor, for which malignancy could be an underlying cause [5].

There are several established risk factors for OSCC. Tobacco, betel quid, and alcohol are predominant risk factors for OSCC [2]. The oral microbiome is one of the major causes of chronic inflammation which facilitates increased cell proliferation, mutagenesis, oncogene activation, and angiogenesis, all of which lead to OSCC progression [6]. The oral microbiome is composed of bacteria, fungi, archaea, and viruses, that interact with each other and show diversity [7]. There is some evidence suggesting that oral microbiota have a role in the development of OSCC [7]. Chattopadhyay et al*.* [8] showed that detecting alterations in oral commensal microbial communities is a potential diagnostic tool for OSCC.

The most likely causes of oral malodor are oral disease, respiratory disease and volatile foodstuffs [5]. Sometimes oral malodor originates from a variety of microbial degradation products, with volatile sulfur compounds (VSCs) being the most common [9]. Common oral VSCs include hydrogen sulfide (H_2_S), methyl mercaptan (CH_3_SH) and dimethyl sulfide ((CH_3_)_2_S). VSCs play an important role in oral malodor [9], and they are mainly produced by Gram-negative anaerobes [10].

Exhalation analysis for lung and breast cancer has been evaluated, and a significant effect of a breath biomarker has been shown [11, 12]. Saalberg et al*.* [11] reported on some useful biomarkers for early lung cancer detection that showed promise for development of a lung cancer screening device. Additionally, Li et al*.* [12] verified the diagnostic value of four exhaled straight aldehydes as early diagnostic biomarkers for breast cancer. Some studies have suggested that analysis of exhaled breath based on mass spectrometry (MS) can be used as a diagnostic method for head and neck cancer [13–15]. In addition, van de Goor et al. conducted analysis using a handheld electronic nose and mentioned the possibility of the device becoming a portable noninvasive diagnostic tool for head and neck cancer [16]. However, exhalation analysis of OSCC patients using simple gas chromatography (GC) has not been conducted much.

Many methods have been developed to measure oral malodor [17]. Exhaled gas analysis devices can be broadly described in two categories, pattern recognition devices with sensors and MS techniques [18]. Among these, GC–MS has long been considered the gold standard for breath VSC analysis [18]. This system has both high sensitivity and semi-specificity for sulfur compounds. However, GC systems are large, expensive, require trained operators, and have long run times. Recently, simple GCs were developed and widely used for quantitative measurement of oral malodor by improving upon the disadvantageous aspects of traditional GCs while maintaining accuracy [17]. Also, the simple GC system is not large and is less expensive. Moreover, sample handling is easier, and trained operators are not required [19]. These new simple GCs will be useful in clinics to detect oral malodor and pathologic oral conditions.

In a study of periodontal disease patients, halitosis was caused by inflammation from periodontal disease, and the exhaled gas of these patients indicated that, hydrogen sulfide and methyl mercaptan had a high concentration among other VSCs [20]. Furthermore, the ratio of CH_3_SH to H_2_S was eight times higher in periodontal disease patients than in healthy people [20]. Although OSCC is different from general periodontal disease, it can also be accompanied by an inflammatory reaction and bad breath. Moreover, OSCC can already be initially detected due to the presence of bad breath [5].

The purpose of this study is to investigate whether the exhaled breath test using a simple GC system is effective for diagnosing OSCC by conducting a comparative analysis of exhaled gas between OSCC and healthy control groups.

## Methods

### VSC analysis with simple GC and collection of samples

VSCs analysis was performed with the Twin Breasor II™ (iSenLab, Seoul, Korea), a simple GC system. This simple GC system was calibrated with standard VSC gases (RIGAS Co., Daejeon, Korea) for five cycles [21]. After the device had warmed up for several minutes, sample air was collected using a mouthpiece and Teflon tubing. VSCs, which are the components of bad breath in oral exhaled gas, are separated into two kinds of gases, H_2_S and CH_3_SH, by gas chromatography using a semiconductor gas sensor.

All subjects came to the hospital following an eight-hour fast in the morning and underwent a simple GC test. No mouth washing or tooth brushing was performed before the test. In the OSCC group, the examination was performed before oral cancer treatment, including surgery, chemotherapy, or radiotherapy. All subjects were allowed to breathe in through the mouth for three minutes and kept their mouths closed while breathing through the nose to incubate the mouth-air sample. The subjects were instructed to relax their chin, close their lips with their mouth slightly open, and then gently bite the wrinkled part of the straw with their lips so that the area around the straw was sealed by the closed lips to minimize VSC contamination. If the lips were opened, ambient air could enter, causing erroneous measurements. After inserting the straw into the mouth, the subjects were instructed to breathe freely through the nose. The measurements for two and half minutes were displayed on the device monitor, from which the concentrations of H_2_S and CH_3_SH were calculated. The individual gas concentrations were then measured and analyzed. The measurement time was about 2 min and 30 s, and the units of measurement were ng/10 ml and ppb.

### Measurement of tongue coating score

The total area and thickness of tongue coating were inspected and recorded by one trained clinician. The area was recorded as a score of 0–3 (0, no tongue coating; 1, tongue coating covering less than 1/3 of the tongue dorsum; 2, tongue coating covering 1/3–2/3 of the tongue dorsum; and 3, tongue coating covering more than 2⁄3 of the tongue dorsum). Thickness was recorded as a score of 0–2 (0, no tongue coating; 1, thin tongue papillae visible; and 2, thick tongue papillae visible). The final tongue coating score was calculated by multiplying the area score by the thickness score [19].

1 $${\text{area}}\;{\text{score}} \times {\text{thickness}}\,{\text{score}} = {\text{tongue}}\;{\text{coating}}\;{\text{score}}\;\left( {{\text{range}}\;0{-}{6}} \right)$$

### Human subjects

Two subject groups were studied: 50 healthy controls (HC) and 50 OSCC patients. The OSCC group included patients aged 20–95 years who were diagnosed with OSCC through a histologic biopsy. The demographic data for the OSCC patients included sex, age, cancer location and staging, treatment modality (operation or chemotherapy), and medical history. The HC group was made up of healthy volunteers with no medical history of malignancy. In addition, HC subjects were those with clinically significant healthy gingiva characterized by the absence of bleeding on probing, erythema and edema, patient symptoms, and attachment and bone loss [22]. Informed consent was obtained from each subject at the time of enrollment. The study was approved by the Institutional Review Board of Seoul National University Dental Hospital (IRB No.: CRI18008).

### Statistical analysis

All statistical analyses were performed using R software, version 4.0.2 (Vienna, Austria, available at: http://www.R-project.org/). Independent two-sample t-tests were used to analyze the difference in exhaled gas concentration between the two groups (HC and OSCC). Kruskal–Wallis one-way analysis of variance (ANOVA) tests and t-tests were used to detect significant differences among various factors, such as location, TNM stage, and treatment modality in the OSCC group with “moonBook” and “UsingR” packages. Receiver operating characteristic (ROC) curves were constructed to assess the sensitivity, specificity, and respective area under the curve (AUC) with a 95% confidence interval (CI) for OSCC detection using the exhaled gas with “multipleROC” and “psych” packages. ROC curves were also constructed with the concentrations of H_2_S, CH_3_SH, and total sulfur and the ratio of CH_3_SH to H_2_S. A new variable was created based on an equation using the H_2_S, CH_3_SH, and ratio values obtained by binary logistic regression to optimize sensitivity and specificity. An ROC curve was drawn with this new variable, and the sensitivity, specificity, and AUC values were calculated. Two-tailed *p* < 0.05 was considered significant.

## Results

### Comparison between HC and OSCC

In the HC (n = 50) and OSCC (n = 50) groups, the male to female ratios were 27:23 and 24:26, respectively, and the differences were not significant. The OSCC group was older than the HC group, and there were no differences in smoking habits or tongue coating scores between the two groups (Table 1).

**Table 1:** Summary of demographic data

	Groups (N = 100)		
	HC (n = 50)	OSCC (n = 50)	*p*
Male	27 (54.0%)	24 (48.0%)	0.689
Female	23 (46.0%)	26 (52.0%)	
Age (mean ± SD, years)	36.3 ± 8.8	60.3 ± 15.0	< 0.001
Smokers	8 (16.0%)	11 (22.0%)	0.610
Tongue coating score (mean ± SD)	1.2 ± 1.5	1.6 ± 1.5	0.217

**Fig. 1:**
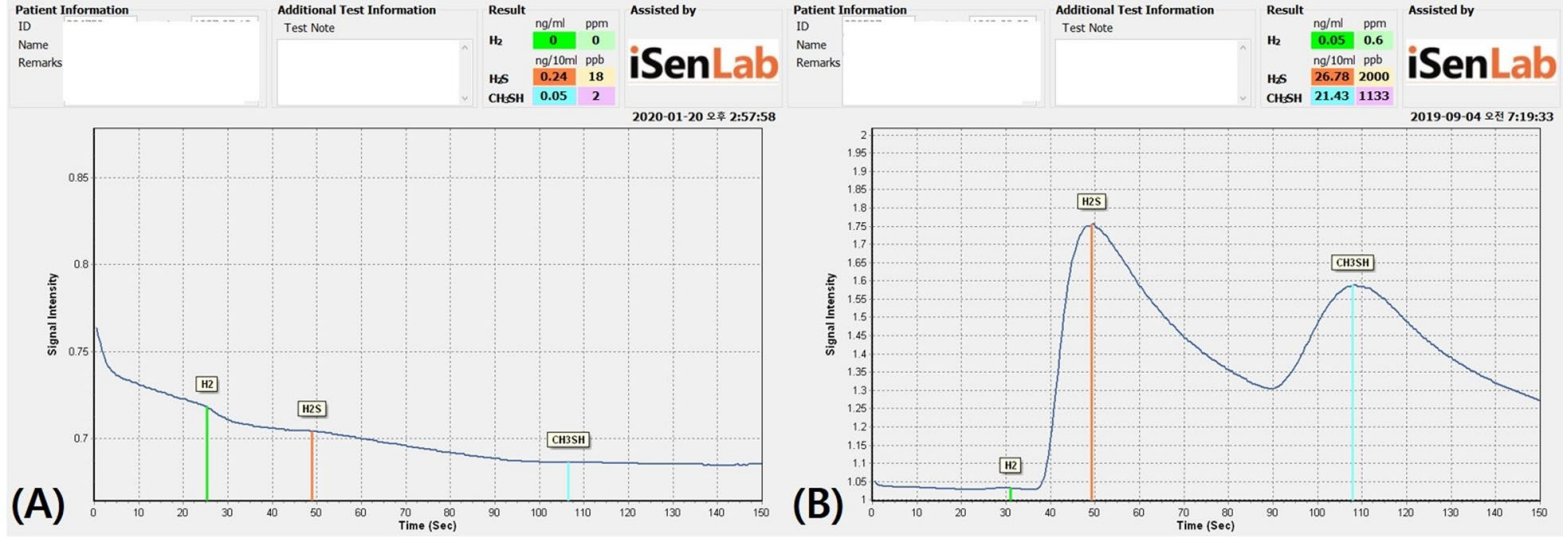
Simple gas chromatogram of an exhaled breath sample from **A** a healthy person and **B** an oral cancer patient

**Fig. 2:**
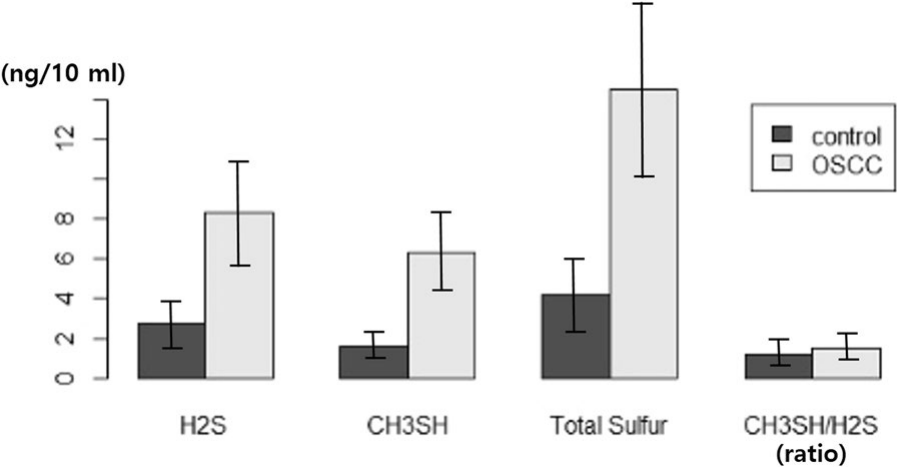
Volatile sulfur compound concentrations with a 95% confidence interval in exhaled breath from two groups (healthy control and oral squamous cell carcinoma)

The Twin Breasor II™ measured the concentrations of H_2_S and CH_3_SH in ng/10 ml and ppb units and constructed a graph of these values to illustrate the broad trends (Fig. 1). Both H_2_S and CH_3_SH were significantly higher in the OSCC group than in the HC group. The total sulfur concentration was also higher in the OSCC group, and there was no significant difference in the ratio of Ch_3_SH to H_2_S between the two groups (Table 2 and Fig. 2).

**Table 2:** Summary of collected concentrations (mean ± SD) of volatile sulfur compounds

	Groups (N = 100)		
	HC (n = 50)	OSCC (n = 50)	*p*
H_2_S (ng/10 ml)	2.7 ± 4.4	8.3 ± 9.6	< 0.001
CH_3_SH (ng/10 ml)	1.6 ± 2.5	6.3 ± 7.3	< 0.001
Total sulfur (ng/10 ml)	4.2 ± 6.7	14.5 ± 15.9	< 0.001
Ratio (CH_3_SH/H_2_S)	1.2 ± 2.6	1.5 ± 2.5	0.541

### Exhaled gas analysis of the OSCC group

In the OSCC group, various factors associated with exhaled GC, such as location, TNM stage, and type of treatment (surgery versus chemotherapy), were analyzed. The primary sites of oral cancers were divided into tongue, gums, and buccal cheek, and VSCs concentrations had no significant difference between the primary sites. Additionally, there was no difference in the concentration of sulfur compounds according to TNM staging. On the other hand, patients who received chemotherapy had significantly higher concentrations of hydrogen sulfide and total sulfur compounds than those who did not (Table 3, Additional file 1).

**Table 3:** Multi-variable analysis of exhaled gas in the oral squamous cell carcinoma group (mean ± SD)

		H_2_S (ng/10 ml)	*p*	CH_3_SH (ng/10 ml)	*p*	Total sulfur (ng/10 ml)	*p*	Ratio (CH_3_SH/H_2_S)	*p*
Location	Tongue (n = 21)	8.6 ± 9.6	0.400	5.4 ± 5.5	0.113	14.0 ± 13.8	0.224	1.7 ± 3.3	0.875
	Gums (n = 23)	6.8 ± 9.1		5.6 ± 7.8		12.4 ± 15.9		1.4 ± 2.0	
	Buccal cheek (n = 6)	12.8 ± 11.8		12.1 ± 9.7		24.9 ± 21.1		1.1 ± 0.6	
TNM stage	I (n = 10)	4.2 ± 6.8	0.464	3.1 ± 3.5	0.897	7.3 ± 9.6	0.614	1.2 ± 1.1	0.253
	II (n = 10)	11.2 ± 10.7		10.7 ± 9.0		21.9 ± 17.2		3.0 ± 4.8	
	III (n = 7)	8.7 ± 11.3		6.1 ± 8.6		14.8 ± 19.3		1.8 ± 2.6	
	IV (n = 23)	8.7 ± 9.7		5.8 ± 6.9		14.4 ± 15.9		0.8 ± 0.7	
Surgery	No (n = 5)	12.7 ± 11.9	0.288	8.4 ± 7.2	0.508	21.0 ± 18.4	0.341	0.9 ± 0.5	0.136
	Yes (n = 45)	7.8 ± 9.4		6.0 ± 7.4		13.8 ± 15.6		1.6 ± 2.6	
Chemotherapy	No (n = 40)	6.9 ± 9.2	0.036	5.4 ± 7.2	0.082	12.2 ± 15.4	0.037	1.7 ± 2.8	0.050
	Yes (n = 10)	13.9 ± 9.5		9.9 ± 7.1		23.8 ± 14.9		0.7 ± 0.5	

### ROC curves for OSCC diagnosis

**Fig. 3:**
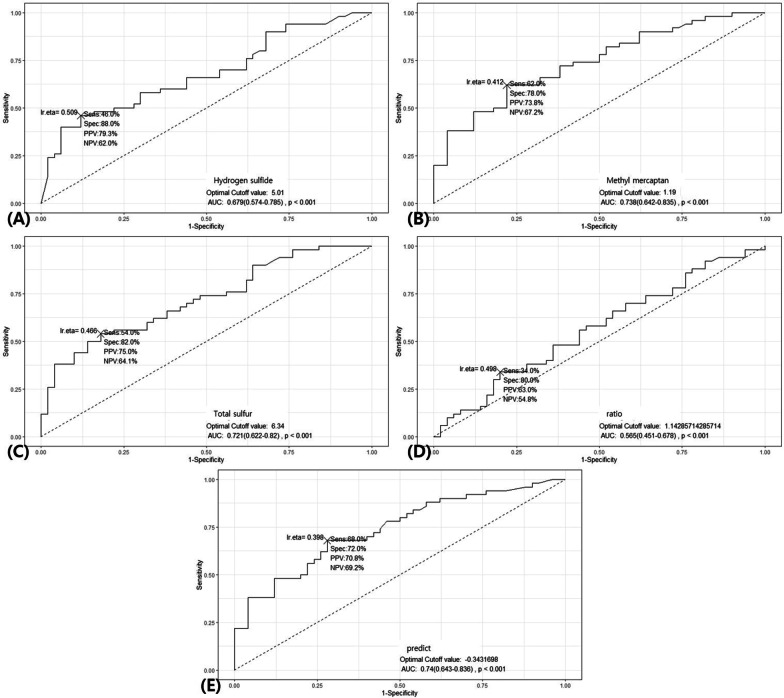
ROC curves for the diagnosis of oral cancer using volatile sulfur compounds: **A** hydrogen sulfide, **B** methyl mercaptan, **C** total sulfur, and **D** the ratio of CH_3_SH to H_2_S. **E** A new variable calculated from logistic regression was utilized, and the ROC curve was drawn

H_2_S, CH_3_SH, total sulfur concentration, and the ratio of CH_3_SH to H_2_S were each evaluated as potential diagnostic variables using ROC curves (Fig. 3) and yielded the following AUCs, respectively: 0.679, 0.727, 0.721, and 0.565 (*p* < 0.001). The sensitivity and specificity values are represented in Fig. 3. To optimize sensitivity and specificity, we created a new variable by binary logistic regression based on an equation using H_2_S, CH_3_SH, and CH_3_SH/H_2_S. The new variable is described with the equation below.

2 $${\text{predict}} = - 0.75193 + {\text{H}}_{2} {\text{S}}*0.03539 + {\text{CH}}_{3} {\text{SH}}*0.16512 + 0.04704*{\text{ratio}}$$

The AUC value for the new variable was 0.740, which was slightly higher than when a single variable was used, and the sensitivity and specificity were 68.0% and 72.0%, respectively.

## Discussion

Both H_2_S and CH_3_SH were significantly higher in the OSCC group than in the HC group. There was no difference in tongue coating score or CH_3_SH/H_2_S between the two groups. According to a previous study, both H_2_S and CH_3_SH were higher in those with periodontitis, with CH_3_SH/H_2_S was more than three times higher than the control group [20]. Periodontal pathogenic microorganisms increased the production of methyl mercaptan in periodontitis patients [20]. In our study, the exhaled breath concentration of gaseous sulfur compounds in OSCC patients was similarly high compared to that of periodontitis and halitosis patients, but the pattern in CH_3_SH/H_2_S was different. Patients with OSCC might be more susceptible to halitosis due to poor oral hygiene, but the pattern is different than that caused by periodontitis, suggesting that exhaled gas analysis can be a potential marker for OSCC diagnosis.

Potential breath biomarkers have also been studied in breast cancer [12, 23]. Pathway analysis revealed that increased glycolysis, lipogenesis, and production of volatile organic metabolites indicated metabolic alterations associated with breast cancer [12]. There are also many factors of oral cancer that alter volatile organic metabolites. The existence of a viable microbiome in deep parts of oral cancer favors the hypothesis that bacteria survive in the tumor microenvironment [8]. Differentiating oral cancer characteristics from bad breath caused by general periodontitis can be a potential diagnostic method for oral cancer.

Accumulated tongue coating instigates bad breath [19]. It might also affect VSC concentrations, but in our results, there was no significant difference in tongue coating score between the two groups. Therefore, it can be said that changes in VSC concentrations in the exhaled gas of OC patients are not related to tongue coating.

Various non-invasive diagnostic methods have been devised supplemental to invasive tissue biopsy. Salivary protein in saliva is a known biomarker [24]. Another study reported that oral brush biopsy can also be used as a non-invasive biomarker for oral cancer [25]. Non-invasive detection methods, such as GC of exhaled breath, have been devised for other pathologic diseases, not just oral cancer. Cellulitis and abscess on the head and neck area have biomarkers of inflammation, including alcohols, aldehydes, and hydrocarbons in exhaled gas [26]. Additionally, saliva incubation can be a promising method for diagnostic discrimination [26]. Because oral tumors can change the oral environment, metabolic investigations of the oral biofilm, oral cancer, and saliva could contribute to accurate diagnostic techniques and, thus, safe and effective treatment for oral and systemic diseases [27].

We found that exhaled breath could be a potential biomarker for diagnosis of oral squamous cell carcinoma by analyzing the concentrations of H_2_S and CH_3_SH. However, there are many other chemical compositions in exhaled breath. Gruber et al. said that ethanol, 2-propenenitrile, and undecane could be potential biomarkers for head and neck tumors [13]. In addition to volatile compounds, exhaled gas also includes other compounds, such as isoprostanes, polypeptides, nucleic acids, lipid mediators, chemokines, and cytokines [14]. Therefore, if we analyze various other molecules of exhaled breath, we will be able to find more volatile biomarkers and produce better results.

The AUC value for the new variable constructed from binary logistic regression was 0.740 (95% CI: 0.643–0.836, *p* < 0.001), and the sensitivity and specificity were 68.0% and 72.0% respectively. These results were similar to those of a study using a portable handheld electronic nose [16], and slightly lower than the results of a study using a selected ion flow tube mass spectrometer [15]. As such, exhaled breath analysis techniques include electric nose, gas chromatography, proton transfer reaction mass spectrometry, and selected ion flow tube mass spectrometry. Accordingly, the number of detected VSCs differs across techniques, as well as the reported sensitivity and specificity of the biomarkers [18]. These results did not demonstrate high accuracy compared with a previous study [12], and are not sufficient to indicate that a simple GC test can be used alone to diagnose oral cancer. However, it can be a valuable non-invasive diagnostic test that can be used as an adjunct prior to more invasive examination. In the future, if the composition and pattern of exhaled gas can be incorporated into the diagnostic approach for oral cancer, it can be a powerful diagnostic tool.

In lung cancer, volatile gases can also play an important role as potential biomarkers. In a study comparing the exhaled breath of head and neck cancer and lung cancer patients, various volatile biomarkers were different by group [28]. In some advanced oral cancers, metastasis to the cervical lymph nodes or lungs occurs [1]. Moreover, because radiotherapy is often used in advanced oral cancer, the halitosis caused by radiotherapy increases, and changes in exhaled breath components can occur [29]. Therefore, exhaled breath analysis in advanced oral cancer requires the consideration of many variables. If exhaled breath analysis is conducted on locally advanced oral cancer that has metastasized to the cervical lymph node or lung, it can be recognized as an important biomarker for recurrent or metastatic oral cancer.

Exhaled gas analysis is non-invasive. With only a simple instrument, the test can be performed quickly and efficiently, and results can be viewed almost immediately. The Twin breather II™ instrument is portable, and sample handling is easy. Trained operators are not required, and it offers similar accuracy to traditional GC [17]. Because early oral cancer and precancerous lesions are difficult to diagnose and often do not show symptoms, many patients already have advanced oral cancer at first diagnosis. Tissue biopsy, the most accurate diagnostic method, is an invasive method that is difficult to perform and takes 1–2 weeks to obtain results. With the Twin breather II™, the exhaled gas analysis test can be performed as a non-invasive preliminary test for oral cancer prior to invasive examinations.

When bad breath worsens for no known reason, VSC concentrations can be checked through a simple GC test, and if the increasing pattern is different from the general periodontitis disease pattern, an oral tumor etiology is a reasonable suspicion. This comparative analysis of exhaled gas between oral squamous cell carcinoma and healthy controls demonstrated a significant difference in VSCs between the two groups.

Although we could see a significant difference in exhaled gas concentration between the OSCC group and the HC group using the easy and portable simple GC, the device we used could only detect H_2_S and CH_3_SH. Other volatile organic compounds, such as formaldehyde, sevoflurane, benzyl cyanide, coumarin, and benzothiazole, can have carcinogenicity [15]. Therefore, if more diverse compounds and their patterns can be detected easily, the diagnostic ability can be further improved. In future research, the reliability of exhaled breath analysis can be increased by comparing the diagnostic ability with other diagnostic tools, such as visual examination.

## Conclusions

Both hydrogen sulfide and methyl mercaptan concentrations were higher in the exhaled breath of OSCC patients, but there was no significant difference in the ratio between the two (CH_3_SH/H_2_S). The ability to detect oral cancer through exhaled H_2_S and CH_3_SH gas and their ratio had an AUC value of 0.740 and sensitivity and specificity values of 68.0% and 72.0%, respectively. These results suggest that exhaled gas analysis via simple GC can potentially serve as an accessory non-invasive method for oral cancer diagnosis.

## Supplementary Information

**Additional file 1**: Raw data containing the collected concentrations of volatile sulfur compounds.

## Data Availability

The datasets used or analyzed during the study are available from the corresponding author upon reasonable request.

## Abbreviations

VSCs: Volatile sulfur compounds
OC: Oral cancer
HC: Healthy controls
AUC: Area under the curve
OSCC: Oral squamous cell carcinoma
MS: Mass spectrometry
GC: Gas chromatography
ROC: Receiver operating characteristic
SD: Standard deviation
